# Effects Following Intracerebroventricular Injection of Immunosuppressant Cyclosporine A On Inhibitory Avoidance Learning and Memory in Mice

**DOI:** 10.22086/gmj.v0i0.1044

**Published:** 2018-08-11

**Authors:** Hamid Reza Banafshe, Mohsen Mohsenpour, Abolfazl Ardjmand

**Affiliations:** ^1^Physiology Research Center, Kashan University of Medical Sciences, Kashan, Iran; ^2^Department of Pharmacology, Kashan University of Medical Sciences, Kashan, Iran; ^3^Department of Physiology, Kashan University of Medical Sciences, Kashan, Iran

**Keywords:** Avoidance Learning, Calcineurin Inhibitor, Cyclosporine A, Mice

## Abstract

**Background:**

Protein phosphatase-2B or calcineurin (CN) is the main phosphatase and a critical regulator of cellular pathways for learning, memory, and plasticity. Cyclosporine A(CyA), a phosphatase and peptidyl-prolyl cis/trans isomerase inhibitor, is a common immune suppressant extensively used in tissue transplantation. To further clarify the role of CN in different stages of learning and memory, the aim of the present study was to evaluate the role of CyA in an inhibitory avoidance (IA) model in mice.

**Materials and Methods:**

Using intracerebroventricular (ICV) injection of different doses of CyA (0.5, 5, and 50 nM) at different periods (pre-/post-training and pre-test), the effect of the drug was evaluated in a step-down IA paradigm. The latency of step-down (sec) was considered a criterion for memory performance.

**Results:**

The pre-training injections of CyA (0.5, 5 nM), however not of 50 nM, impaired IA learning acquisition. The post-training injection of high-dose CyA (50 nM) impaired memory consolidation. The pre-test ICV CyA injection did not impair memory retrieval; the ICV injection of CyA caused no change in locomotion.

**Conclusion:**

These findings suggest that CyA selectively interferes with acquisition, retention, but not retrieval, of information processing in mice. Given the crucial role of CN in common signaling pathway of memory performance and cognition, it could be a probable therapeutic target in the treatment of a wide variety of neurological conditions involving memory.

## Introduction

Cyclosporine A(CyA), an immunosuppressant commonly used in clinical settings to prevent graft rejection [1], is considered a modern drug for organ transplantation [2]. CyA, rapamycin, and FK506 belong to a group of drugs known as immunophilins or neurophilins [3]. CyA has diverse and numerous neuronal effects, e.g., neurogenesis [4, 5], neuroprotection [6-8], transmitter action, inhibition of nitric oxide mechanism, inhibition of cAMP, and morphine effects. CyA acts via a group of peptidyl-prolyl cis/transisomerizes (PPIases) or cyclophilins and protein phosphatases (PP2B), also called calcineurin(CaN) [9].CaN is the main substrate for converting the different forms of memory [10], and cyclophilins have roles in protein folding and neuronal signal processing [11].

Many researchers argue that in disclosing the functions of proteins involved in learning and memory processes, the importance of memory-impairing proteins is similar to that of memory-enhancing ones [12].

The pivotal role of Ca+-dependent phosphatase, CaN, in learning and memory is well known[13].

Also, the role of CaN in synaptic plasticity of any type has been reported [14].

A large corpus of studies on the effects of CyA on learning and memory using different models shows the following contradictory results: pre-train, but not post-training central injection of CyA in spatial learning model, resulted in memory enhancement [15].

Other experiments on chick using intracerebral CyA and inhibitory avoidance (IA) models revealed learning was impaired [16]. In genetic experiments, over-expressed CaN impaired long-term memory in mice [10], and the use of antisense DNA against CaN resulted in long-term potentiation in rats [17].

The role of PP2B with CyA in chick memory formation was studied. CyA via the inhibition of CaN impaired the later and final stages of memory formation [18].

The separate unilateral administration of the drug in the right and left hemisphere [14] showed a sort of laterality for CyA effects.

Given the ubiquitous distribution of CaN in nervous system neurons, CaN alters the functions of diverse types of proteins and controls the level of neuronal excitation. Brain CaN content is more than 1% of the whole protein [19].

Hence, the rationale that CaN is ubiquitously distributed in nervous system deserves examining the hypothesis that the agents blocking its function may be considered as the main candidate substrates for studying the pathology of neurotoxicity occurring on using calcineurin blockers [20] or relevant pathologies. Moreover, besides the value of CyA in clinical settings [2], working on brain phosphatases has advantages that interest neuroscientists. With similar reasoning, like with other adaptable systems, phosphatase systems seem to be appropriate candidates for those interested in studying brain mechanisms and different types of learning and memory.

The role PP2B (CaN) plays in learning and memory processes has attracted great attention because of its critical role, the drugs inhibiting these activities (e.g., CyA), and the contradictory results on the effect of CyA on learning and memory.

Against this background, the main objective of the study was to evaluate the impacts of intracerebroventricular (ICV) injection of immunosuppressant CyA on IA learning and memory in mice.

## Materials and Methods

### Animals

A total of 140 adult male albino mice (weight 25–30 g) were housed in Plexiglas cages (8 mice in each cage) inside a well-ventilated room at 22±2 ºC with a 12-hour light/12-hour dark cycle. A 10% mortality of the animals was observed during the surgery and treatment periods. The animals had free access to standard food and water.

All experiments were carried out between 0800–1500 hours and each experiment group included nine mice.

The Ethics Committee for Animal Studies (Kashan University of Medical Sciences, Kashan, Iran) approved all ethical procedures of the study (# 9169), which was conducted in accordance with the International Guidelines for Laboratory Animal Care and Use (NIH publication #85-23, 1985 revision).

### Surgery and Cannula Implantation

The animals were anesthetized with an intraperitoneal (ip) cocktail of a ketamine/xylazine (50 mg/kg and 5 mg/kg) and were held in a stereotaxic frame (Stoelting Co., Illinois, US) with flat skull position. After a midline incision on the skin of the skull and the retraction of underlying periosteum, a stainless steel guide cannula was implanted above the third dorsal ventricle. Stereotaxic coordinates were: midline, 0.5 mm posterior to bregma, 3 mm ventral, based on the atlas of Franklin and Paxinos [21].

The cannula was fixed to the skull with dental cement and jewelry screw. After the surgery,

one stainless steel stylet was put into the guide cannula for maintaining patency before drug microinjections [22]. Once the drug was injected, the animals were allowed a one-week recovery period from surgery to clear the effect of anesthetic drugs.

### Drugs and Microinjection

CyA was acquired from Novartis (Basel, Switzerland). All drugs were dissolved with DMSO and ethanol (99%) and prepared to the required volume and concentration. Grounded in previous experiments, a mixture of the non-toxic concentration of DMSO [3] plus ethanol was used. Also, the applied doses of CyA were selected according to relevant studies [14, 15]. The ICV microinjections of the fresh drugs in a volume of 5 µL/mice were administered using a 27-gauge injection cannula (1 mm longer than the guide cannula to extend 1 mm beyond).

The injection cannula was fixed with a polyethylene tube to a Hamilton Syringe (5µL). The ICV microinjections were administered over 60 sec, and the injection cannula was left in the cannula for an extra 60 sec to help diffuse the drug from the tip [23].

The pre-/post-train or pre-test ICV microinjections of the drugs were conducted after a 5 min interval with respect to the training or testing.

### Step-down Inhibitory Avoidance (IA) Setup

The step-down IA setup consisted of a Plexiglas box (40×30×30 cm) with a floor of steel bars (0.75 cm in diameter with a spacing of 1 cm). A platform (dimensions 4×4×4 cm) in the central bottom area of the floor was provided. On placing the animal on the platform, its natural tendency is to get down on the floor bars. When the animal was on its four paws, an electric shock (frequency 1Hz at 15 v for 15 sec) using a stimulator was administered on the animal’s limb. The latency time of getting down from the safe platform (step-down latency) was considered an index for memory retrieval [24].

This task involved two stages of training and testing. In the training session, animals were gently placed on the platform in the middle of the device, and a chronometer recorded the delay in coming down from the platform. After descending the platform, the animal immediately received an electric shock for 15 sec. In the case of a successful IA response, the animal was removed from the device and immediately received a post-training injection of the drug.

At the test session conducted 24 h after the training phase, no shock was delivered to the animal. At this session, each animal was gently placed on the platform again and the time delay for coming down from the platform was recorded as a memory retrieval index. In these experiments, the cut off time was 300 sec [24].

### Open Field Apparatus

Open field apparatus was used to ensure the effectiveness of the drug on locomotion. The setup consisted of a rectangular black-colored chamber connected to a CCD camera and its dedicated software in a PC (Technique Azma, Tabriz, Iran). The locomotor activity of the animals by open field was studied on test day, immediately after completing the memory tests. The total distance traveled by each animal (cm) in 5 min was specified for each mouse as an index of spontaneous locomotion.

### Experiment Design

#### Experiment 1:

This experiment examined the effects of pre-train ICV injections of saline or vehicle (DMSO+ethanol) on IA memory performance. Two groups of mice were used. On the training day, the control and vehicle control groups received pre-training injections of saline (5µL/mouse) and DMSO+ethanol, respectively. On the test day, the control and vehicle control groups received pre-test saline (5 µL/mouse) and DMSO+ethanol treatment, respectively.

#### Experiment 2:

The experiment examined the effects of pre-train ICV injections of CyA. Four animal groups were used. The vehicle control animals received pre-train and pre-test injections of vehicle (5µL/mouse).

The other three groups received pre-train (5 µL/mouse) CyA with different doses (0.5, 5, and 50 nM). On the test day, all the groups received pre-test vehicle (5 µL/mouse) treatment.

#### Experiment 3:

The experiment examined effects of post-train ICV injections CyA. Four animal groups were used. The vehicle control group received post-train and pre-test injections of vehicle (5 µL/mouse). The remaining three groups received post-train CyA (5 µL/mouse) of different doses (0.5, 5 and 50 nM). On the test day, all the groups received pre-test vehicle (5 µL/mouse) treatment.

#### Experiment 4:

This experiment examined the effects of pre-test ICV injection of CyA on IA memory performance in mice. Four animal groups were used. During the training session, all groups received vehicle (5 µL/mouse) after training. On the test session, the experiment groups received pre-test injections of CyA with different doses (0.5, 5 and 50 nM/mouse) and post-train injections of the vehicle.

### Histological Verification

After terminating the experiments, each mouse was anesthetized with an overdose of chloroform and methylene-blue (1 µL, 1%) slowly infused into the lateral ventricle and perfused transcardially with a phosphate-buffered saline solution (pH=7.4). The mice were decapitated, and the brain removed and preserved in formalin (10%). After several days, the fixed brain samples were sliced, and the sites of injection were verified, based on Paxinos and Franklin atlas [21]. Only data obtained from animals with correct cannula implantations were included in the analyses.

### Data Analysis

Data were evaluated using parametric/non-parametric t-tests and one-way analysis of variance (ANOVA). Further analyses for paired group comparisons were conducted with post hoc Tukey’s and Holm-Sidak tests. P-value<0.05 was considered the significance level.

## Results

### Effect of Pre-train Injection of Saline or Vehicle on IA Learning Performance

**Figure-1:**
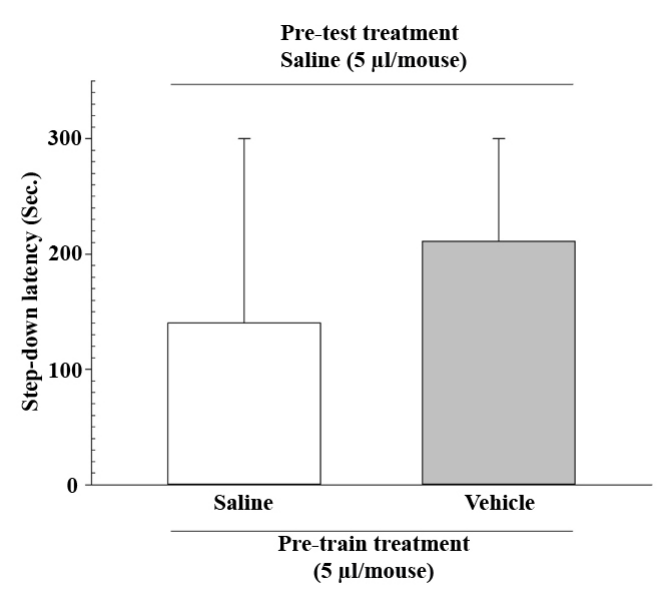
Effects of pre-train ICV injections of saline or vehicle on inhibitory avoidance performance. The administration of saline (5 μl/mouse, ICV) or vehicle were done 5 min before the training. Each bar represents median±quartile of mice per group (n=9)

Figure-1 shows the effects of pre-train administration of saline or vehicle on IA learning performance in mice. The Mann-Whitney U statistical analysis demonstrated no significant differences between the control (saline) and vehicle control groups (P>0.05).

### Effect of Pre-train ICV Injection CyA on IA Learning Acquisition

**Figure-2:**
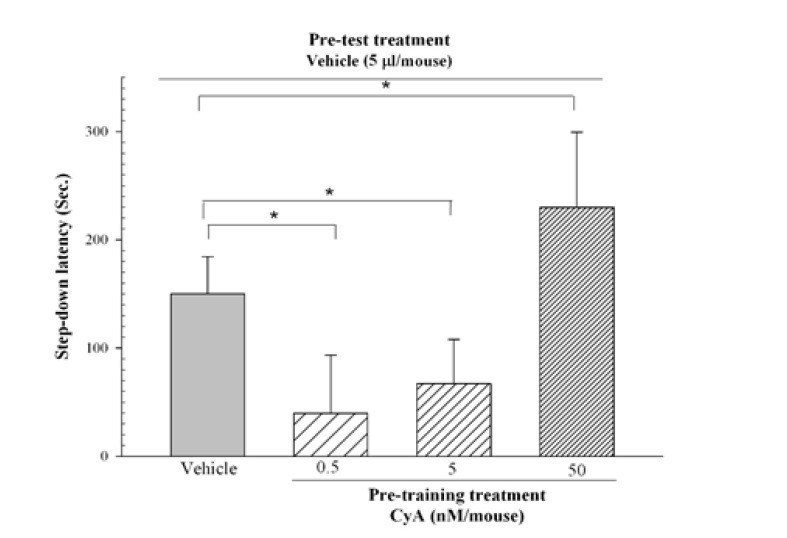
Effects of pre-train ICV injections of vehicle or CyA on inhibitory avoidance learning acquisition. The administration of vehicle (5 μl/mouse, ICV) or different doses of CyA (0.5, 5, and 50 nM/ mouse) were done 5 min before the training. Each bar represents mean±SD of mice per group. *P-value <0.01 compared to the vehicle-vehicle control group (n=9)

Figure-2 shows the effects of pre-train administration of CyA on IA learning acquisition in mice. One-way ANOVA revealed significant differences between the groups (F_3, 32_=17.85, P<0.001); however, post hoc Holm-Sidak test showed that pre-train CyA (0.5, and 5 nM/mice, ICV) impaired learning acquisition. While CyA (50 nM/mice, ICV) enhanced the IA learning acquisition compared with the vehicle control that received the pre-train vehicle.

### Effect of Post-train ICV Injections of CyA on IA Memory Consolidation

**Figure-3:**
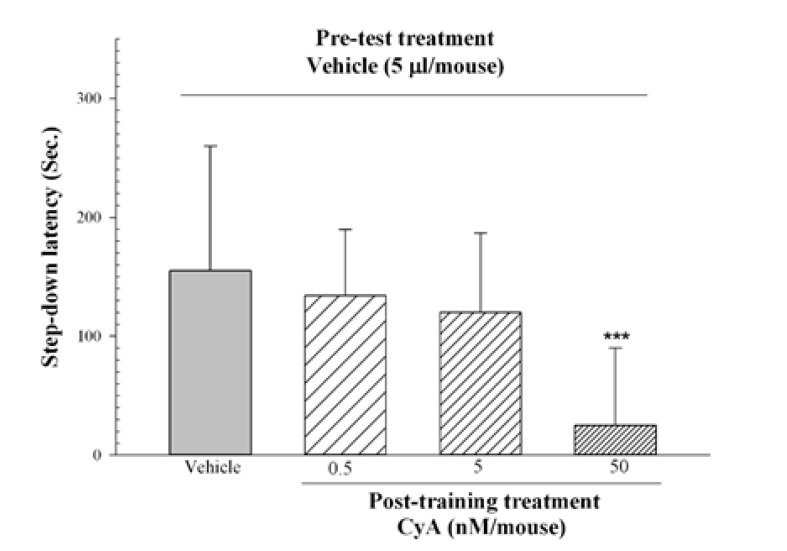
Effects of post-train ICV injections of vehicle or CyA on inhibitory avoidance learning consolidation. The administration of vehicle (5 μl/ mouse, ICV) or different doses of CyA (0.5, 5, and 50 nM/ mouse) were done immediately after the training. Each bar represents mean±SD of rats per group. ***P-value <0.001 compared to the vehicle-vehicle control group (n=9)

Figure-3 shows the effects of post-train injection of CyA on IA memory consolidation. One-way ANOVA revealed a significant difference between the groups that received different doses of CyA (F_3, 32_=6.47, P<0.002). However, post hoc Tukey’s test also revealed that post-training CyA (50 nM/mice, ICV) impaired the IA memory consolidation compared with the vehicle control group (P<0.002).

### Effect of Pre-test ICV Injections of CyA on IA Memory Retrieval

**Figure-4:**
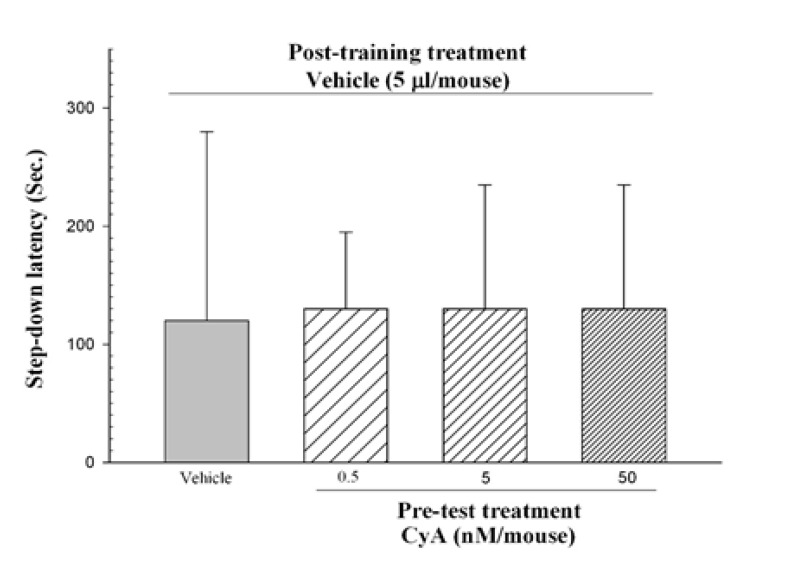
Effects of pre-test ICV injections of vehicle or CyA on inhibitory avoidance learning retrieval. The administration of vehicle (5 μl/rat, mouse) or different doses of CyA (0.5, 5, and 50 nM/ mouse) were done 5 min before testing. Each bar represents median±quartile of mice per group (n=9)

Figure-4 reveals the effects of pre-test administration of CyA on IA memory performance. Kruskal-Wallis nonparametric ANOVA showed no significant difference between the groups (F_3, 32_=0.0623, P=0.996).

### Effect of CyA on the Mice Locomotor Activity

**Figure-5:**
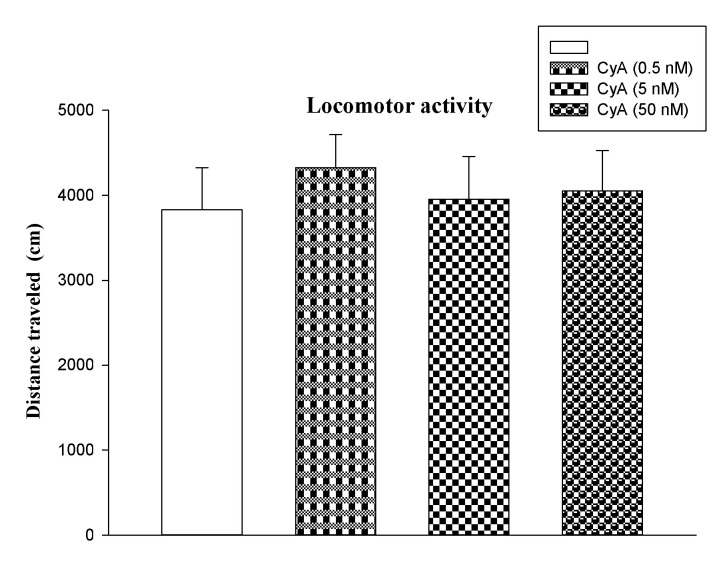
Effects of ICV injections of vehicle or CyA on locomotion. The locomotion was performed on the second day in an open field after the completion of the memory testing. Each bar represents mean±SD of rats per group (n=9)

Figure-5 reveals the effects of CyA on IA memory retrieval. Kruskal-Wallis nonparametric ANOVA showed no significant difference between the groups (F_3, 32_=0.816, P=0.494).

## Discussion

The objective of the present study was to explore the effects on IA learning and memory in mice following ICV injection of immunosuppressant CyA. The researchers observed some novel and apparently unexpected effects. The main findings of the study were as follows.

Pre-train administration of CyA (0.5, and 5 nM/mouse/ICV) impaired IA learning acquisition in animals; however, CyA (50 nM/mouse, ICV) enhanced the IA learning acquisition compared with the control that received pre-train vehicle treatment. Post-training administration of different doses of CyA (0.5, 5 and 50 nM/mouse/ ICV) impaired IA memory consolidation, with the maximum significant effect seen on CyA 50 nM. Also, the pre-test CyA at all applied doses had no significant effect on IA memory performance. Finally, the findings suggest that CaN inhibition does not play a significant role in locomotor activity in the mouse open field model.

CaN as the ubiquitous and main initial substrate for learning and memory, and plasticity [25] is a calcium/calmodulin-dependent phosphorylase that, through the activation of PPIase [19, 26], has a modulatory role in synaptic function [19].

Consistent with the preliminary findings of this study, in IA experiments on chick memory, impairment was observed at far lowest and highest doses beyond the effective dose [27]. The present study showed that low-dose CyA has no effect on acquisition [28]. Hence, the question why impairment was seen in this study only with the low to medium doses of CyA may be explained by the fact that a low–medium dose of CyA has the potential of incomplete inhibition of PP2B. In Shaw *et al*. study, the bilateral intra-hippocampal injection of CyA at low to high doses resulted in a partial to complete inhibition of CaN [15]; or a medium dose of CaN inhibition in the lateral ventricles of mice resulted in a similar effect [29]. Other researchers argue that in both constitutive and stimulated calcineurin levels, the acquisition is the most sensitive stage of learning and memory [30]. Also, findings of the present study suggest an enhancing effect for post-training CyA (50 nM). The observed enhancing effect is in line with the results, indicative of an initial enhancing effect of the PP2B blockade, in which the enhancing effect of memory by PP2B inhibition begins in the initial post-training periods [31] using the most effective dose that inhibit PP2B[27].

Based on different molecular approaches suggested by other researchers, the apparent contradictory effects of enhancement and impairment that might be seen by phosphatase inhibition in some learning and memory studies may relate to the nonspecific effects of CaN inhibitors[32].

Other researchers also propose an inverted U-shaped inhibition for PP2B action [28]. Likewise, a number of researchers reported such a U-shaped effect for some drugs affecting memory performance [13].

While at present we cannot provide a convincing explanation from our experiments, it should be added that application of phosphatase inhibitors in learning and memory processes may result in some controversial localized/systemic or lateralized/bilateral outcomes [16], some of which are given below. For example, on peripheral injection of CyA, no effect was seen on retention of learning [33], while its central injection resulted in inhibition of inhibitory learning[18]. Many studies show that apart from the time of treatment, the most effective dose of CyA causes retention impairment [27]. Also, as another example, in some experiments, the unilateral injection of CyA[14] demonstrated effects different from its bilateral injection [15]. Away from these findings, it may be concluded that since the present study used the third ventricle injection of CyA, the observed effect has been attributed to the combinational unilateral effects of CyA [16].

Memory formation usually consists of a period of protein folding, processed by PPIase, the main substrate for CyA inhibition. CyA is also considered a PPIase inhibitor of cyclophilins, the key element in protein folding [34, 35]. As cyclophilins are generally available in the brain, and except for their co-expression with CaN, no specific functions are attributed to them [36]; they probably perform significant functions non-relevant to their CaN inhibition. Besides their role in memory processing and with the consideration that CaN have roles in protein folding, the effects of CyA on CaN and PPIase may be superimposed.

Alternatively, PPIase has a role in the final stage of memory processing in some animals (e.g., chick). It is promising that the phase of protein synthesis is required for long-term memory formation in the chick and other species [27].

Some effects of CyA inhibition are probably independent of CaN inhibition[37].

These studies prove the hypothesis that the mechanism of protein folding by cyclophilin has a significant and unknown role in cell signaling [35, 38] and, considering the critical role of translation in information processing of memory, the memory-impairing effect of CyA via the inhibition of PPIase is inevitable [39].

Also, a type of the delayed effect for PPIases enzymes aiding the protein folding is seen as well [35], where the impairing effect by PPIase inhibition begins with a delayed period relative to post-training [16, 18]. In agreement with our current findings, some studies report that CaN probably have no function in the terminal stages of memory performance [16].

As CyA probably inhibits other groups of PPIases, there is doubt CyA inhibits PP2B. Based on evidence where post-training CyA was used to inhibitPP2B, CaN has no function in the initial stages of memory processing as it was revealed that some drugs are known to inhibit CaN [18].

Although among the experiments carried out in the laboratory for this study, no clear evidence of memory enhancement was observed at every dose or time. Hence, we believe that the lack of these enhancing effects may also to some extent be attributed to ceiling effects in the protocol used, which permit no space for improvement beyond the control level.

Furthermore, given the failure of CyA to show similar effects, a sort of non-specific effect [40] is implausible to account for the observed effect, although some types of CaN, not sensitive to CyA, are also reported [20].

As the unexpected effect of CyA in the present study is consistent with the notion that the drug may have more than one effect, this proposition may be evaluated using agents that can selectively inhibit PPlase with no effect on CaN activity, along with studies measuring both PPIase and CaN activities in the brain at various time intervals following CyA application.

Taken together, the mechanisms induced by CaN inhibitor in these experiments remain unclear. However, while recall of long-term memory can be sustained in the presence of CaN inhibitor (CyA), that of the short-term to intermediate memory may be disrupted.

Experiments for evaluating the effects of drugs on the brain’s higher functions, like the one this study presented, advance the matter of “drug repositioning” for CyA that open new horizons for the use of phosphate inhibitors in the treatment of cognitive disorders in future.

## Conclusions

This study reveals that the ICV injection of CyA interferes with acquisition, retention, but not retrieval, of IA performance in mice. Considering the pivotal role of CN in common signaling pathway of higher functions such as memory and cognition, CN could be a probable therapeutic target in the treatment of some neurological disorders involving memory in future.

## Conflict of Interest

All authors of this paper declare no conflict of interest.
